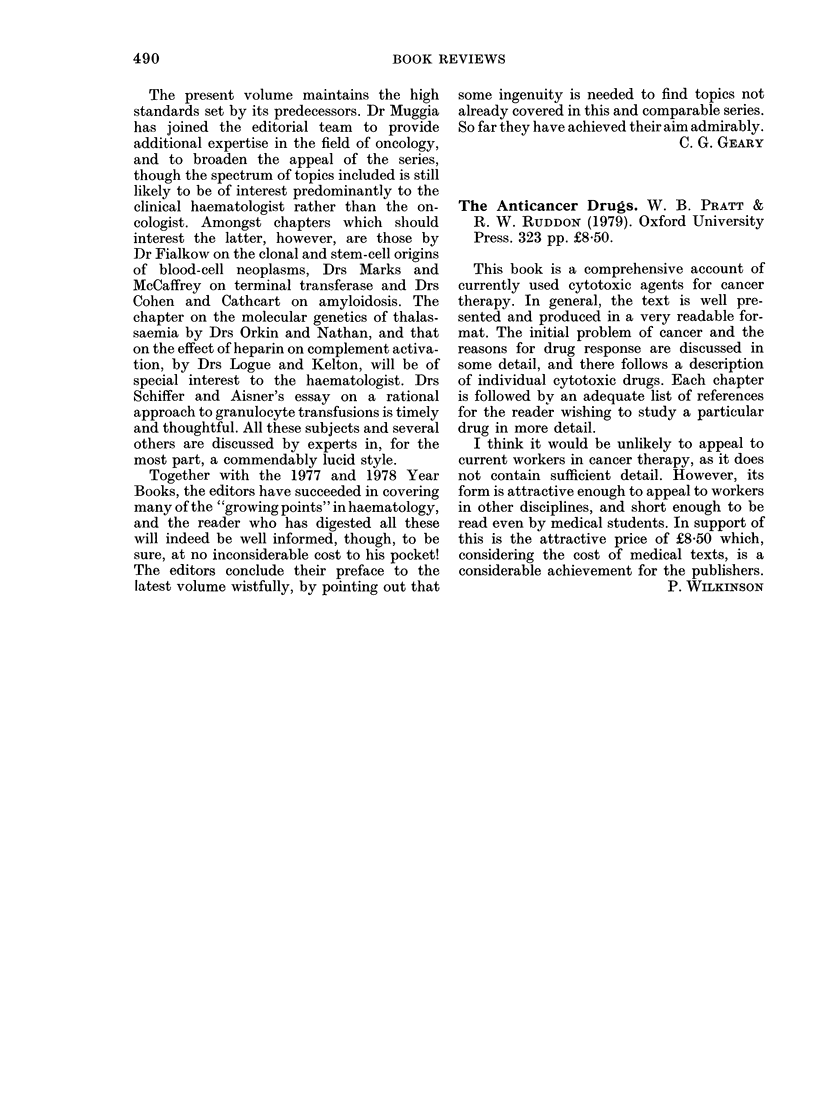# The Anticancer Drugs

**Published:** 1980-09

**Authors:** P. Wilkinson


**The Anticancer Drugs.** W. B. PRATT & R. W. RUDDON (1979). Oxford University Press. 323 pp. £8·50.

This book is a comprehensive account of currently used cytotoxic agents for cancer therapy. In general, the text is well presented and produced in a very readable format. The initial problem of cancer and the reasons for drug response are discussed in some detail, and there follows a description of individual cytotoxic drugs. Each chapter is followed by an adequate list of references for the reader wishing to study a particular drug in more detail.

I think it would be unlikely to appeal to current workers in cancer therapy, as it does not contain sufficient detail. However, its form is attractive enough to appeal to workers in other disciplines, and short enough to be read even by medical students. In support of this is the attractive price of £8·50 which, considering the cost of medical texts, is a considerable achievement for the publishers.

P. WILKINSON